# Risk Indicators of Peri-Implant Diseases in Public and Private Clinics: A Multicenter Study

**DOI:** 10.1155/2024/7061682

**Published:** 2024-08-17

**Authors:** Mahdi Kadkhodazadeh, Reza Amid, Omid Amirinasab, Omid Amirbandeh, Anahita Moscowchi

**Affiliations:** ^1^ Dental Research Center Research Institute for Dental Sciences School of Dentistry Shahid Beheshti University of Medical Sciences, Tehran, Iran; ^2^ Department of Periodontics School of Dentistry Shahid Beheshti University of Medical Sciences, Tehran, Iran

## Abstract

**Methods:**

Patients examined during postloading maintenance visits were included in this study. The presence of peri-implant mucositis, peri-implantitis and several patient- and implant-related independent variables was recorded. Statistical analysis was performed using the logistic regression analysis. The odds ratios (OR) of the potential association between each variable and the occurrence of peri-implant diseases were evaluated.

**Results:**

Among the 114 participants with 403 implants, peri-implantitis was found in at least one implant of nine individuals (7.89%), and a total of 13 implants were affected by peri-implantitis (3.22%). The univariate regression analysis revealed a statistically significant association between arch (OR = 4.81; 95% CI = 1.27–31.36) and soft tissue thickness (OR = 4.07; 95% CI = 1.33–13.73) with the occurrence of peri-implantitis. The multivariate analysis confirmed the significant impact of soft tissue thickness (OR = 3.60; 95% CI = 1.16–12.24).

**Conclusion:**

The occurrence of peri-implant diseases can be influenced by various factors. However, in order to accurately identify risk indicators, it is necessary to conduct long-term prospective studies.

## 1. Introduction

Implant therapy has dramatically changed oral rehabilitation since its introduction [1], with an improved survival rate over the last decade [2, 3]. However, adverse events such as biological complications including mucositis and peri-implantitis may compromise the treatment outcome.

Peri-implantitis is a biofilm-induced pathological condition affecting peri-implant tissues, characterized by inflammation in the mucosa around dental implants and subsequent progressive bone loss [4, 5]. Peri-implantitis is not uncommon among patients with dental implants [6], and when it is not properly managed, its progression follows a nonlinear and accelerating pattern, which may be related to the involved microorganisms, the host's defense mechanism, and the absence of periodontal ligament [7, 8].

Regular maintenance visits of professional care are the best way to prevent peri-implantitis and prompt intervention when indicated. Therapeutic approaches proposed for peri-implantitis have limited predictability, and even when disease resolution is achieved, recurrence may occur in the long term [9, 10]. Consequently, its prevention becomes of utmost significance, mainly based on managing peri-implant mucositis [11]. This strategy should undoubtedly consist of the control of modifiable risk factors [4, 12]. Therefore, the recognition of risk factors and indicators of peri-implant diseases is the focus of current research in implant dentistry.

Different factors may influence the occurrence of peri-implantitis, some of which are patient-related such as compliance with oral hygiene habits, regular maintenance visits, smoking, and history of periodontal disease. Still, other parameters including soft tissue condition, prosthetic design, timing protocol of implant placement, and loading duration are contributing factors in peri-implant health and disease [4].

Several studies have reported data on the prevalence and risk indicators of peri-implant diseases in university settings [13, 14, 15]. The use of samples with regular maintenance visits may hinder the true analysis of the disease prevalence and risk indicators [16]; hence, there is a need for further studies in different settings and with representative samples [17, 18]. This study aimed to estimate the prevalence of peri-implant diseases and their potential risk indicators among patients rehabilitated with dental implants.

## 2. Materials and Methods

This multicenter study was conducted following the Declaration of Helsinki, after approval by the Ethics Committee of Shahid Beheshti University of Medical Sciences, Tehran, Iran (IR.SBMU.DRC.REC.1398.216). Patients who received at least one implant were consecutively included during their visits for any dental complaint to the Shahid Beheshti School of Dentistry and three private clinics in Tehran, Iran (February 2022 to September 2022). This study was designed taking into consideration the strengthening the reporting of observational studies in epidemiology (STROBE) statement guidelines for reporting cross-sectional studies. Written informed consent was obtained from all participants before their enrollment.

### 2.1. Sampling Procedures

The determination of the sample size was conducted by considering the assumption that the occurrence rate of peri-implantitis was equivalent to 36.9% as reported in the study conducted by Gunpinar et al. [19]. The required sample size was 99 for the absolute precision of 10% in estimating the prevalence with 95% confidence and considering the potential loss of 10%. Participants with the following characteristics were excluded: (1) consuming medications that affect bone metabolism; (2) antibiotic treatment in the last 2 months before the examination; (3) not interested in participating in the study; (4) pregnancy or breastfeeding in women; and (5) less than 12 months since the suprastructure placement.

### 2.2. Data Collection

Patients' records were screened to retrieve their smoking status, history of periodontal disease and diabetes, date of implant insertion and loading, the timing of implant placement [20], recall visit intervals, number of implants, and prosthetic design. The following parameters were recorded for each implant (clinical examinations were performed using Williams Colorvue™ Probe (Hu-Friedy Mfg. Co., Chicago, IL, USA)):

- Marginal bone loss (MBL) was measured using digital software (Scanora, KaVo, Germany). The long cone paralleling method was utilized to calculate the MBL up to the most coronal bone-to-implant contact on both mesial and distal surfaces. To take into account potential radiographic distortion, implant length was used for calibration. For each implant, the worst value between mesial and distal bone levels was considered for case definition.
- Probing depth (PD) on four points (mesial, distal, midbuccal, and midlingual). For each implant, the worst value was considered for case definition.
- Bleeding on probing (BoP)/suppuration based on a binary scale (presence/absence).
- O'Leary plaque index [21].
- Implant site position (maxilla or mandible and anterior or posterior).
- Implant connection (platform-matched/platform-switched).
- Prosthetic design (splinted/nonsplinted).
- Keratinized tissue width (KTW).
- Soft tissue biotype (thin or thick) based on the probe transparency through the soft tissue [22].
- Soft tissue recession (STR) if the abutment surface was exposed to the mouth.
- Vestibular depth (VD). The details of the examinations were provided in the previous studies [23, 24].

All clinical examinations were conducted by experienced clinicians (MK, OA, OA, and AM), who were previously calibrated. The level of agreement between the examiners was evaluated using a sample of 10 patients (interrater agreement for marginal bone level = 0.89; soft tissue phenotype = 90.7%).The intrarater reproducibility was determined by the evaluation of the same 20 randomly chosen implants twice within a minimum of a 24-hr interval, which revealed an agreement of 0.93. Professional maintenance was provided for all patients; implant therapy was done whenever necessary, and oral hygiene instructions were reinforced or modified if required.

### 2.3. Peri-Implant Health and Disease Case Definitions

Recall visits at intervals ≤6 months were considered regular recall appointments. The absence of BoP and no PD ≥ 6 mm were defined as healthy implants (if there was bone loss extending beyond the remodeling of the crestal bone, without any BoP and no PD ≥ 6 mm, it was considered clinically stable and healthy). Peri-implant mucositis was diagnosed in the case of BoP/suppuration with no PD ≥ 6 mm and MBL ≥ 3 mm. The threshold for the definition of peri-implantitis was the presence of bleeding and/or suppuration on gentle probing and bone levels ≥3 mm apical of the most coronal portion of the intraosseous part of the implant in the presence of PD ≥ 6 mm [5]. KTW was defined as narrow (0–2 mm), medium (3–4 mm), and wide (≥5 mm). VD was also categorized as shallow (0–5 mm), medium (6–10 mm), and deep (≥11 mm). Plaque index was classified as <30% and ≥30% [13].

Frequencies and percentages were utilized to present descriptive statistics. The occurrence of peri-implant mucositis and peri-implantitis was documented at both the implant and patient levels. A patient was counted as a case in the prevalence calculation of mucositis and peri-implantitis if one or more of their implants met the criteria for mucositis and peri-implantitis, respectively. Therefore, a patient could be counted as a case in both groups.

### 2.4. Data Analysis

The analysis involved examining risk indicators for peri-implant mucositis and peri-implantitis. Univariate logistic regression analysis was conducted for each independent variable individually to determine factors associated with peri-implant mucositis and peri-implantitis. Factors with *P* values < 0.05 were chosen for the multivariate logistic regression. To avoid multicollinearity, Spearman's correlation analysis was used. Peri-implant mucositis and peri-implantitis were the binary dependent variables, and the other variables were independent. Odds ratio (OR) estimates and 95% confidence intervals (95% CI) were retrieved from the intercept of each factor. Generalized estimating equations (GEE) analysis was conducted for peri-implantitis, taking into account the fact that some patients had multiple implants. Patient IDs were used as the subject variable in the analysis. The predictors that demonstrated a significant relationship in the univariate analysis were subsequently included in the model. The statistical software SPSS (IBM, Version 26.0, Armonk, NY) was utilized for all analyses, and statistical differences with *P* values < 0.05 were considered significant.

## 3. Results

A total of 114 patients (39 males and 75 females) with 403 bone level dental implants were screened for this study. The mean age of the patients was 50.14 ± 12.7 years, ranging from 24 to 79 years. The number of implants per patient ranged from 1 to 15 implants. A total of 10 participants (8.8%) were smokers, and eight patients (7%) were diabetic. The median postloading follow-up was 30 (12–225) months.

The descriptive statistics are summarized in Tables 1 and 2. Clinically, 177 (43.9%) implants presented BoP. The mean PD was 2.58 ± 1.09 mm (3.01 ± 0.99 mm for peri-implant mucositis; 5.32 ± 1.94 mm for peri-implantitis). The mean gingival recession was 0.05 ± 0.22 mm.

Patient-level prevalence of peri-implant mucositis and peri-implantitis was 50.9% and 7.89%, respectively. A total of 52 patients had mucositis with no implants with peri-implantitis, three patients had implants with peri-implantitis and no mucositis, and six patients had implants that showed both mucositis and peri-implantitis characteristics. At the follow-up examination, it was found that 40.7% of implants had peri-implant mucositis and 3.2% had peri-implantitis.

Table 3 depicts the ORs and 95% CIs for each patient-based factor associated with peri-implant mucositis and peri-implantitis. Based on the univariate regression analysis, the number of dental implants was found to have a significant association with peri-implant mucositis (OR = 2.5, 95% CI = 1.31 to 5.75; *P*=0.026). No patient-level variable showed a significant association with the occurrence of peri-implantitis.

The implant-based variables associated with peri-implant mucositis and peri-implantitis are summarized in Table 4. The variables that were found to be significant through univariate regression analysis were then used in multivariate regression analysis. The results showed that several independent variables were significantly associated with peri-implant mucositis. These variables include immediate implant placement (OR = 2.35; 95% CI = 1.43 to 3.88; *P* < 0.001), having implant-supported partial restorations (OR = 2.33; 95% CI = 1.52 to 3.58; *P* < 0.001), having splinted restoration (OR = 1.78; 95% CI = 1.12 to 2.87; *P*=0.016), and treatment at the public center (OR = 2.00; 95% CI = 1.32 to 3.05; *P*=0.001). Implants in the mandible (OR = 4.81; 95% CI = 1.27 to 31.36; *P*=0.042) and having thin soft tissue biotype (OR = 1.78; 95% CI = 1.33 to 13.73; *P*=0.015) were significantly associated with peri-implantitis. In the multivariate regression model using significant variables, the development of peri-implant mucositis was found to have significant associations with immediate implant placement (OR = 2.00; 95% CI = 1.19 to 3.41; *P*=0.009), having implant-supported partial restorations (OR = 1.78; 95% CI = 1.09 to 2.92; *P*=0.019), and treatment at the public center (OR = 2.34; 95% CI = 1.50 to 3.68; *P* < 0.001). Thin soft tissue biotype showed a significant association with peri-implantitis in the multivariate regression analysis (OR = 3.60; 95% CI = 1.16 to 12.24; *P*=0.028). GEE analysis for peri-implantitis also showed a significant association with soft tissue thickness (OR = 1.28; 95% CI = 0.25 to 2.30; *P*=0.015).

## 4. Discussion

This study, which was carried out at various centers, aimed to investigate the prevalence of peri-implant diseases and the factors that indicate a potential risk for developing these diseases. The findings revealed that 7.89% of patients had peri-implantitis, and the arch and soft tissue biotype were identified as significant risk indicators for peri-implantitis.

Implant survival, which is typically reported in studies, can overestimate implant outcomes. Implant success is used to evaluate the condition and function of the implant. Therefore, it could offer a better measure if specific success criteria are universally defined, accepted, and used. However, criteria for implant success have not been used consistently, and no commonly accepted definition of implant success has been established. Depending on the criteria used, the rates of implant success reported in studies can vary substantially. The use of stringent criteria would produce implant success rates lower than those determined using less strict criteria. A systematic review evaluating the prevalence of peri-implant disease reported that peri-implant mucositis and peri-implantitis occurred in 19%–65% and 1%–47% of cases, respectively [25].

In this study, peri-implantitis was diagnosed based on the 2017 World Workshop on Periodontal and Peri-implant Diseases and Conditions criteria. The patient- and implant-based prevalence of peri-implantitis in this study was estimated at 7.89% and 3.22%. Weinstein et al. [18] conducted a multicenter study to evaluate the prevalence of peri-implantitis. The reported prevalence was similar to the present study, which affected 4.03% of patients and 1.20% of implants. Nonetheless, based on the defined bone loss threshold, the reported prevalence could be different [13, 26, 27]. Matarazzo et al. [13] evaluated a Brazilian population with no initial data and detected peri-implantitis in 39.8% of individuals and 20.5% of implants. However, they used a 2 mm MBL as a threshold for peri-implantitis diagnosis. The effect of the potential impact of a change in classification criteria on the prevalence of peri-implantitis was confirmed in a cross-sectional study by Shimchuk et al. [28]. They reported a nearly 50% reduction in the prevalence of peri-implantitis at both patient and implant levels, using the 2017 World Workshop on Periodontal and Peri-implant Diseases and Conditions criteria compared with the 2015 classification. Romandini et al. [14] also reported an implant-based prevalence of 27.9% using a ≥ 2 mm threshold of bone loss, while peri-implantitis with MBL ≥ 3 mm was present in 12.4% of the studied implants. The present study would yield different results if the criteria were modified to include a combination of BoP/pus and bone loss ≥2 mm. In such a scenario, the prevalence of peri-implantitis at the patient level would be 15.8%, while at the implant level, it would be 8.2%. If the criteria for defining the case were bone loss of 3 mm or more accompanied by BoP or pus, the prevalence of patient- and implant-related peri-implantitis would be 12.3% and 6.9%, respectively. Additionally, if baseline radiographs are obtained after the prosthesis connection, the prevalence rate may differ [17, 29]. Given that baseline radiographs were accessible for all implants placed at the university center during the restoration process, the prevalence was reevaluated by taking into account the presence of BoP, increased PD, and bone loss. As a result of this reassessment, the implant-based prevalence of peri-implantitis would rise from 6.5% to 11.5%.

**Figure 1:**
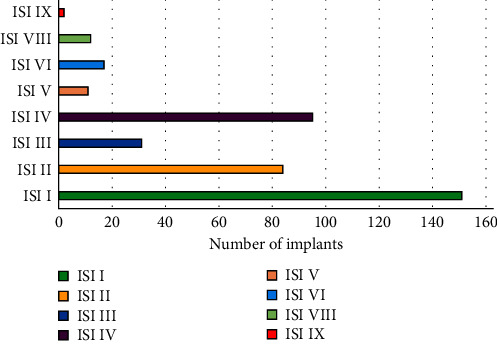
Distribution of implants based on implant success index (ISI).

The wide range of prevalence could be attributed to adopting different study designs, follow-up periods, cutoff thresholds, mean time in function, and sample sizes. Thus, criteria based on the success of implant therapy may be more accurate in interpreting the data of such studies. The implant success index (ISI) was proposed in 2012 to overcome some of these limitations [30]. This index can be used not only to diagnose peri-implantitis but also to determine its severity (Figure 1). A new classification was also proposed to assess peri-implant tissue condition both before and after prosthetic loading of dental implants [31].

In this study, 60% of implants with peri-implantitis functioned for more than 5 years. It should be noted that the high rate of peri-implant mucositis (40.7%) may eventually lead to peri-implantitis in the long term, as a recent systematic review and meta-analysis found that the occurrence of peri-implantitis at the implant level increased from 0.4% within 3 years to 43.9% within 5 years [32]. The regression analysis showed a significant association between peri-implant mucositis with the number of implants, multiple adjacent implants, and splinted restorations. It should be noted that most of the neighboring implants had splinted restorations. All these factors may hinder proper oral hygiene. Therefore, clinicians should be aware of the importance of diagnosing peri-implant mucositis at the earliest stage to prevent its progression to peri-implantitis. The patients should also be convinced about the necessity for regular visits and proper oral hygiene habits to diminish the risk of developing peri-implant diseases.

The regression analysis conducted in this study showed mandible and thin soft tissue as potential predictors of developing peri-implantitis. As the results of different studies are controversial [17, 29], no definite conclusions may be reached concerning either jaw being at a greater risk of developing peri-implantitis. Furthermore, soft tissue parameters are usually not included in the studies on peri-implantitis, or KTW is the only variable in this regard, which could underestimate the importance of soft tissue conditions on the outcome of implant therapy. The results of this investigation are in line with the previous study conducted to assess the effect of soft tissue condition on peri-implant health [23], since it was indicated that the deficient soft tissue condition contributes to a higher rate of BoP, PD, and peri-implantitis. A recent systematic review also confirmed the positive effect of soft tissue augmentation on peri-implant health status [33]. A recent consensus report showed that reduced keratinized mucosal width is associated with increased biofilm accumulation, soft tissue inflammation, patient discomfort, mucosal recession, MBL, and an increased prevalence of peri-implantitis [34]. Less MBL at sites with thicker soft tissue is also documented in the literature [35].

As it has been previously emphasized, VD could play an important role in peri-implant health though it is not addressed properly in the studies conducted on the risk indicators of peri-implantitis. Although the regression analysis did not show a significant association between VD and peri-implantitis, it was significantly related to BoP, PD, and peri-implant STR. The significant association between the arch and KTW with BoP may also be attributed to the shallow VD in the posterior mandible, as the arch and KTW were significantly relevant to VD (*P* < 0.001).

Most of the implants included in the study were found to have cemented restorations, making it inappropriate to compare them with screw-retained prostheses. However, consistent with previous research [36], cemented restorations were associated with favorable outcomes. All implants featured an internal connection. While platform-switched implants have been proposed to have lower MBL [37], no significant differences were observed considering marginal bone level, mucositis, and peri-implantitis between platform-matched and platform-switched implants, aligning with the results of another retrospective study [38].

We used similar case definitions to Matarazzo et al. [13] in terms of independent variables; nevertheless, different risk indicators of peri-implantitis were recognized in either study. It could be attributed to the factors included in the regression analysis. Therefore, the results of the regression analysis may be affected by the number of imputed criteria, the parameters determined as factors or covariates, and the case definition for the categorization of the independent variables.

Similar to any investigation, the present study had some limitations. The inherent drawback of cross-sectional studies is that the temporal link between the outcome and the exposure cannot be determined. In addition, similar to periodontitis, peri-implantitis may have some periods of remission, which could underestimate the actual prevalence of the disease in the cross-sectional studies. The follow-up in the present study was also short for some cases, which may justify the low prevalence of peri-implantitis. Diagnosis based on the follow-up radiographs may underestimate or overestimate the bone loss in some patients depending on the diagnosis criteria. Since old radiographs are not always available in cross-sectional studies, clinicians often use only the current set of radiographs as was done in this analysis. Another noteworthy issue is that some factors such as the clinicians' skill, loading protocol, the reason for tooth loss, and additional therapeutic measures such as augmentation techniques were not assessed in this study, which may also affect the results. For instance, all implants in the present study were placed by periodontists who possess expertise in managing both hard and soft tissues. This factor could potentially contribute to the relatively low occurrence of peri-implantitis.

Case definition can significantly affect both the prevalence and risk indicators of peri-implant diseases. Therefore, it is highly recommended that distinct criteria should be defined not only for the diagnosis of peri-implant mucositis and peri-implantitis but also for the categorization of the potential risk indicators. This homogeneity would help to compare the results of different studies and draw a definitive conclusion.

## 5. Conclusion

In the present study, the patient- and implant-level prevalence of peri-implantitis was 7.89% and 3.22%, respectively. It should be noted that the case definition can influence the rate of prevalence. Some factors may impact the occurrence of peri-implant diseases; however, long-term prospective studies are needed to detect risk indicators more accurately.

## Figures and Tables

**Table 1:** Patient-related factors evaluated in this study.

Factor	*N* = 114	Percentage
	*n*	
Gender		
Female	75	65.8
Male	39	34.2
Age		
<60 years	82	71.9
≥60 years	32	28.1
History of periodontal disease		
Yes	9	7.9
No	105	92.1
Diabetes		
Yes	8	7
No	106	93
Smoking		
Yes	11	9.6
No	103	90.4
Regular recall visits		
Yes	23	20.2
No	91	79.8
Full-mouth plaque index		
<30%	9	7.9
≥30%	105	92.1
Implant number		
<4 implants	76	66.7
≥4 implants	38	33.3
Center		
Public	45	39.5
Private	69	60.5

**Table 2:** Implant-related factors evaluated in this study.

Factor	*N* = 403	Percentage
	*n*	
Timing of implant placement		
Immediate	80	19.9
Late	323	80.1
Site position		
Anterior	74	18.3
Posterior	329	81.7
Arch		
Maxilla	185	45.9
Mandible	218	54.1
Implant connection		
Platform-matched	171	42.4
Platform-switched	232	57.6
Type of prosthesis		
Single	271	67.2
Multiple	132	32.8
Prosthetic design		
Splinted	296	73.4
Nonsplinted	107	26.6
Implant time in function		
<24 months	90	22.3
≥24 months	313	77.7
Keratinized tissue width		
Narrow	187	46.6
Medium	161	40.0
Wide	54	13.4
Gingival biotype		
Thick	286	71.0
Thin	117	29.0
Vestibular depth		
Shallow	151	37.5
Medium	189	46.9
Deep	63	15.6
Center		
Public	139	34.5
Private	264	65.5

**Table 3:** Patient-related potential predictors for the occurrence of peri-implant mucositis and peri-implantitis.

Variable	Peri-implant mucositis			Peri-implantitis		
	Univariate regression			Univariate regression		
	OR	95% CI	*P* value	OR	95% CI	*P* value
Gender						
Male	1.64	0.75 – 3.63	0.213	0.62	0.15 – 2.66	0.503
Age						
≥60	1.35	0.59 – 3.10	0.474	1.40	0.31 – 9.75	0.685
History of periodontal disease						
Yes	3.70	0.84 – 25.65	0.122	0.65	0.10 – 13.01	0.711
Diabetes						
Yes	1.66	0.38 – 8.45	0.499	0.57	0.08 – 11.36	0.620
Smoking						
Yes	1.78	0.50 – 7.15	0.378	0.84	0.13 – 16.43	0.877
Regular recall visits						
No	0.60	0.22 – 1.51	0.286	1.14	0.16 – 5.15	0.873
Full-mouth plaque index						
≥30%	4.21	0.91 – 27.69	0.093	1.51	0.07 – 9.29	0.711
Implant number						
≥4	2.50	1.31 – 5.75	0.026^*∗*^	0.36	0.08 – 1.47	0.153
Center						
Private	0.46	0.21 – 0.99	0.052	1.24	0.29 – 4.98	0.751

OR, odds ratio; CI, confidence interval;  ^*∗*^statistically significant.

**Table 4:** Implant-related potential predictors for the occurrence of peri-implant mucositis and peri-implantitis.

Variable	Peri-implant mucositis						Peri-implantitis					
	Univariate regression			Multivariate regression			Univariate regression			Multivariate regression		
	OR	95% CI	*P* value	OR	95% CI	*P* value	OR	95% CI	*P* value	OR	95% CI	*P* value
Timing of implant placement												
Late	0.42	0.25–0.69	<0.001^*∗*^	0.53	0.31–0.91	0.022^*∗*^	3.04	0.58–55.92	0.287	—	—	—
Site position												
Posterior	0.76	0.46–1.28	0.309	—	—	—	2.76	0.53–50.72	0.332	—	—	—
Arch												
Mandible	0.74	0.44–1.24	0.259	—	—	—	4.81	1.27–31.36	0.042^*∗*^	4.22	1.10–27.69	0.064
Implant connection												
Platform-matched	1.28	0.86–1.92	0.218	—	—	—	1.19	0.32–4.02	0.754	—	—	—
Type of prosthesis												
Partial	2.33	1.52–3.58	<0.001^*∗*^	1.99	1.21–3.31	0.007^*∗*^	0.90	0.24–2.85	0.876	—	—	—
Prosthetic design												
Splinted	1.78	1.12–2.87	0.016^*∗*^	1.54	0.90–2.69	0.116	1.21	0.36–5.48	0.773	—	—	—
Implant time in function												
≥24 months	1.40	0.86–2.31	0.171	—	—	—	3.54	0.68–65.05	0.226	—	—	—
Keratinized tissue width												
Medium	1.44	0.76–2.78	0.262	—	—	—	0.66	0.06–14.4	0.738	—	—	—
Narrow	1.32	0.60–2.16	0.701	—	—	—	2.87	0.53–53.32	0.319	—	—	—
Soft tissue thickness												
Thin	1.05	0.67 – 1.62	0.827	—	—	—	4.07	1.33 –13.73	0.015^*∗*^	3.60	1.16 –12.24	0.028^*∗*^
Vestibular depth												
Medium	0.67	0.37–1.20	0.177	—	—	—	0.43	0.09 –2.24	0.281	—	—	—
Shallow	1.08	0.59–1.96	0.798	—	—	—	0.82	0.21–4.02	0.793	—	—	—
Center												
Private	0.49	0.32–0.75	0.001^*∗*^	0.42	0.27–0.66	<0.001^*∗*^	1.19	0.38–4.46	0.774	—	—	—

OR, odds ratio; CI, confidence interval;  ^*∗*^statistically significant.

## Data Availability

The data underlying this article cannot be shared publicly due to the privacy of individuals who participated in the study. The data will be shared on reasonable request to the corresponding author.
